# Sex-Specific Analysis of Carotid Artery Through Bilateral 3D Modeling via MRI and DICOM Processing

**DOI:** 10.3390/bioengineering12020142

**Published:** 2025-02-01

**Authors:** Pedro Martinez, Jose Roberto Torres, Daniel Conde, Manuel Gomez, Alvaro N. Gurovich

**Affiliations:** 1 Clinical Applied Physiology (CAPh) Lab, The University of Texas at El Paso, El Paso, TX 79968, USAdaconde@utep.edu (D.C.); mgomez26@utep.edu (M.G.); 2 Aerospace Engineering MS Program, New Mexico State University, Las Cruces, NM 88003, USA; polaris@nmsu.edu; 3 Department of Physical Therapy and Movement Sciences, The University of Texas at El Paso, El Paso, TX 79968, USA

**Keywords:** carotid artery, magnetic resonance imaging, anatomical differences, vascular geometry, endothelial shear stress, stroke risk

## Abstract

The present study explores the anatomical differences between sexes of the carotid artery using non-invasive magnetic resonance imaging (MRI) and a DICOM processing protocol. Bilateral three-dimensional models of the carotid artery were constructed for 20 healthy young adults, 10 males and 10 females, in order to evaluate key anatomical landmarks; these include the bifurcation diameter and angle, as well as the internal and external carotid arteries (ICA and ECA) for both sides (left and right). The results show that males exhibit larger bifurcation and ECA diameters, which could indicate reduced endothelial shear stress (ESS). However, as there is no previously observed sex difference in ESS between sexes, compensatory factors might be in play, such as blood pressure. This underscores the interaction between vascular geometry and stroke risk disparities; future research is encouraged to analyze diverse demographics and employ flow modeling techniques to further asses the connection between anatomical differences within a given population and vascular outcomes.

## 1. Introduction

Cardiovascular disease remains the leading cause of mortality worldwide, contributing to nearly 18 million deaths per year, with ischemic stroke as a major cause of premature mortality and long-term disability. In recent years, stroke death rates have exhibited concerning trends, particularly among adults between the ages of 45 and 64, with an increase of 12% between 2019 and 2021 [1]. Even though there is a higher incidence of cardiovascular disease in males than in females, the general incidence of stroke is higher in females than males [2]. In addition, approximately 9 out of 10 ischemic strokes are associated with atherosclerosis [2], which is when fat and cholesterol build up in the arteries or blood vessels, causing the arteries to narrow [3]. Over time, the atherosclerotic plaque can break and bleed, causing an ischemic stroke. Atherosclerotic plaque development is highly associated with anatomical and functional factors, such as bifurcations and endothelial shear stress (ESS) [4,5,6]. Blood flow patterns, which include ESS and the presence or not of turbulence, might be the most relevant factors in the activation of the vascular endothelium, via mechanotransduction and nitric oxide (NO) metabolism, to prevent and treat atherosclerosis [4,5,6].

We have recently studied blood flow patterns [7] and mechanical characteristics [8] of the common carotid artery (CCA) in males and females, finding no differences between sexes at rest or during exercise. However, anatomical differences between sexes, especially bifurcation angles and vessels diameters, are yet to be determined.

Angiography, both in the carotid or coronary arteries, is considered the gold standard for the diagnosis and visualization of intravascular pathologies, particularly coronary artery disease (CAD) and stroke [9,10]. However, as an invasive procedure, there are many risks and complications associated with it, such as allergic or adverse reactions related to ionized-based contrast agents [9], as well as contrast-induced nephropathy, particularly in patients with chronic kidney disease, diabetes, anemia, or hemodynamic instability [11,12]. These limitations increase the demand for non-invasive alternatives [10,13]. In this context, magnetic resonance imaging (MRI) presents significant advantages as it eliminates the need for intravenous contrast agents or ionizing radiation and instead relies on the intrinsic magnetic properties of body tissue and blood and its reaction to external magnetic fields [14]. The focus on advancing non-invasive procedures has allowed MRI technology to produce high resolution images, as well as three-dimensional time-of-flight (TOF) sequences that match the diagnostic capabilities of angiography, making it an indispensable tool for planning medical interventions [15,16].

Another widespread non-contrast MRI technique is phase contrast (PC), which utilizes velocity encoding to measure blood flow and visualize vascular structures, making it a valuable tool for assessing hemodynamics within the carotid arteries; however, these two techniques (TOF and PC) differ in limitations. For instance, TOF excels at acquiring high-resolution structural images, but suffers from saturation effects in slow or turbulent flow regions, while PC provides robust flow visualization, while requiring longer imagine times and being more prone to artifacts caused by motion or pulsation [17].

Multiple studies have validated the accuracy of non-contrast MRI in depicting carotid artery structures. For instance, it has been demonstrated that TOF is in good agreement with digital subtraction angiography (DSA) at assessing carotid stenosis. As for acquisition speed and image resolution, both have been continuously improved by advancements in compressed sensing and parallel imaging, enhancing the overall clinical utility of non-contrast MRI [15,18].

Even though there have been several studies assessing carotid artery differences between males and females [19,20,21], most of these studies focused on atherosclerotic plaque size and distribution. Unfortunately, studies using non-contrast MRI to determine ESS-sensitive geometry associated with ESS-dependent blood flow patterns are, to the best of our knowledge, scarce.

Therefore, the purpose of this study was to determine ESS-sensitive anatomical differences in the carotid artery between sexes using non-contrast MRI and an imaging processing protocol designed in our lab. We used non-contrast MRI and Digital Imaging and Communications in Medicine (DICOM) processing to build bilateral 3D models of the carotid artery. The work presented here employs advanced segmentation and modeling techniques that could have profound implications for planning preventive and therapeutic treatments for vascular diseases.

## 2. Materials and Methods

### 2.1. Participant Characteristics

The present study was reviewed and approved by the Institutional Review Board of The University of Texas at El Paso while adhering to the principles of the Declaration of Helsinki. All the participants provided written informed consent. Twenty apparently healthy young adults, ten males and ten females, between the ages of 18 and 35 were recruited for this study. Exclusion criteria included previous history of cardiovascular, metabolic, or neurological disease, a blood pressure of <120/80 mmHg, and self-reported physical activity levels of more than 150 min per week. The participants were instructed to abstain from food, caffeine, alcohol, non-steroidal anti-inflammatory drugs, and antioxidant supplements for at least 8 h before testing and to avoid exercising for 24 h prior to the study to control for changes in the vasomotor function of the carotid artery. Participants were asked to turn up for an appointment at an imaging center (University Medical Center, Northeast, El Paso, TX, USA) 30 min before their assigned time where they rested for 10 min before assessing their blood pressure, which was obtained using an automated brachial blood pressure cuff (BP760, Omnron Healthcare, Inc., Lake Forest, IL, USA). Then, height and mass were taken using a calibrated stadiometer and scale, respectively (Detecto PHR, Detecto, Webb, MO, USA). Finally, participants had a neck MRI (Magneton Skyra, Siemens, Malvern, PA, USA), and images were stored as DICOM for processing. The neck MRI examination followed previously described parameters, including sparce Time-of-Flight (TOF) of ~4 min, echo time/repetition time of 3.43/21 ms, field of view of 200 × 180 mm^2^, interpolated voxel size of 0.3 × 0.3 × 0.4 mm^3^, and an acceleration factor of 2.5× [22].

### 2.2. Setup of 3D Slicer

The segmentation of the carotid arteries through the software 3D Slicer (https://www.slicer.org/, version 5.4.3) [23] was performed to facilitate the overall modeling. The extensions *SlicerVMTK*, *CurveMaker*, *FiducialsToModelDistance*, *MarkupsToModel* and *PathReconstruction* were added to the software from the extensions tab of the program. The setup utilized for this work employs the Vascular Modeling Toolkit (VMTK) module. This will produce a slice of 0.625 mm of thickness in the sagittal, coronal, and transverse planes of the scans.

### 2.3. DICOM File Compiling

**Figure 1:**
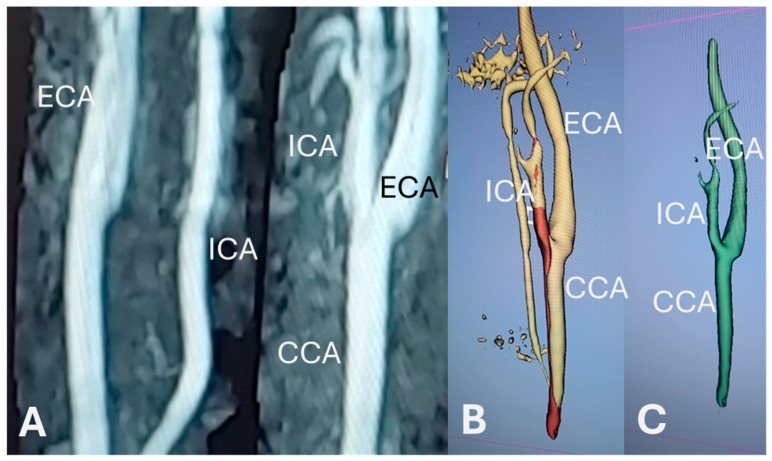
Images from one representative participant. (**A**) DICOM images; (**B**) image on 3D Slicer after vessel segmentation; (**C**) fully processed 3D image. CCA: common carotid artery; ICA: internal carotid artery; ECA: external carotid artery.

The imaging data were stored in CD disks which were later processed via an external CD reader device (Verbatim Extra Slimline CD/DVD Writer). Once processed, several files were obtained, a DICOM folder containing the required data, a visualizer for a low-resolution angiogram, as well as three support files for running the data. After storing all these files into the working environment, the data can be imported to 3D Slicer through a built-in function (Figure 1A).

### 2.4. Vessel Segmentation

After importing the files into 3D Slicer, the segment editor is opened automatically, signaling readiness for segmentation. Each segment, which represents a distinct anatomical structure, must be manually created by being added in the segment editor, then assigning a descriptive name and applying a unique color for visual differentiation. After adding a segment, the opacity range must be defined, the ranges used for these visualizations are between 240.00 and 270.00. Finally, to view the resulting model, the *Masking* menu needs to be opened, and the option *everywhere* must be selected and applied (Figure 1B).

### 2.5. Segmentation Processing

After the vessel segmentation, the data were cleaned out of noise by utilizing the segment editor module, and this was performed carefully, avoiding the deletion of big portions of the areas of interest, such as the common carotid artery (CCA), the internal carotid artery (ICA), and the external carotid artery (ECA) (Figure 1C).

### 2.6. Landmark Determination

**Figure 2:**
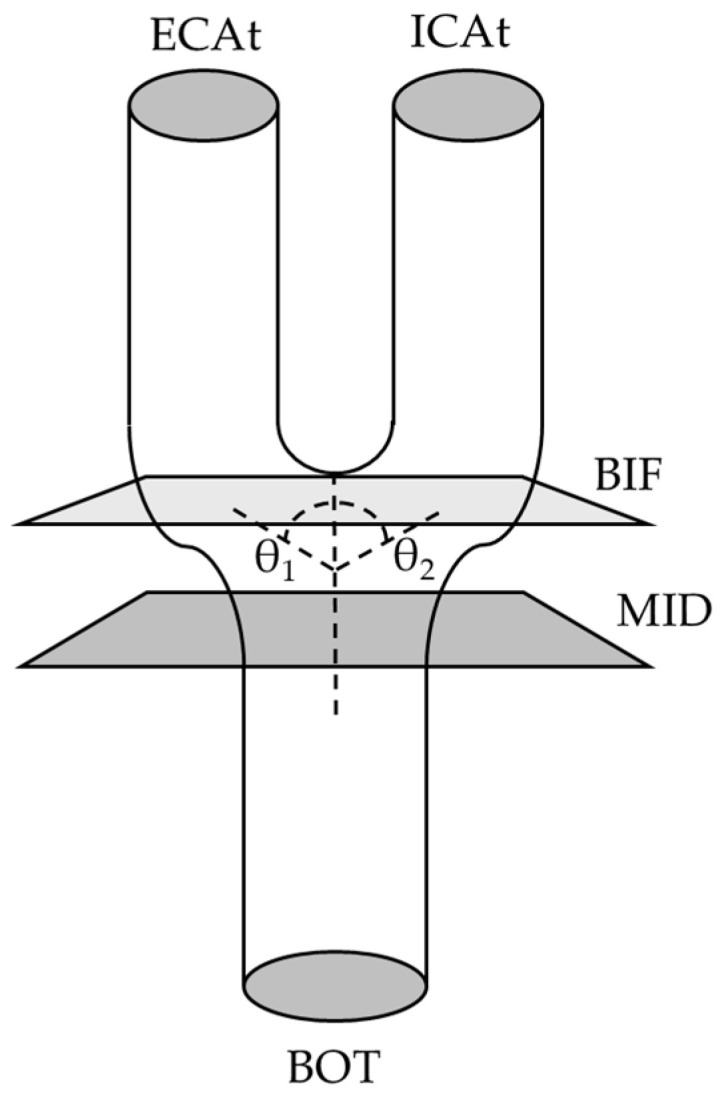
Diagram of the landmarks.

Key anatomical landmarks were identified based on previous studies [24,25,26]. Briefly, we include the midpoint (MID), which determines the center of the model between the superior and inferior portions, the bifurcation (BIF) point, which indicates the point where the CCA is visibly equal in its separation into ICA and ECA and the bifurcation angle, the bottom (BOT), denoting the end portion of the manual detailing of the CCA, and lastly, the top (TOP), referring to the upper limit of the manual detailing of the carotids, for both ECA and ICA, referred to as ECAt and ICAt, respectively. A schematic representation can be found in Figure 2.

#### 2.6.1. Midpoint (MID)

The determination of the diameter at MID was achieved by rotating the 3D specimen toward the transversal plane, manually with the segment editor window, until the BOT of the specimen showed as a circle. Then, moving up through the frames with either the keyboard arrows or the slider until reaching the point where the CCA continues appearing as a fully circular shape just before becoming an ellipse, typically within 3–5 frames below the bifurcation. Each carotid required a unique measurement. MID represents the CCA diameter just before bifurcation.

#### 2.6.2. Bifurcation (BIF) Point and Angle and ECA and ICA Angles

After saving the MID, BIF point (diameter) will be determined by the frame where the CCA ends being a full circle and both ECA and ICA are still connected, and the frame shows an ellipse shape more than two independent circles. The selected frame is highly related to the bifurcation area which varies between carotids; for this reason, anatomical knowledge as well as logical reasoning was needed, while observing all coronal, sagittal, and transversal planes, to determine and appropriate BIF with adequate height and depth. In addition, on the lateral plane, the BIF, ECA, and ICA angles were determined by drawing the CCA midline and ECA and ICA midlines. The sum of ECA angle (θ_1_ in Figure 2) and ICA angle (θ_2_ in Figure 2) will determine the BIF angle.

#### 2.6.3. TOP

The previous process is repeated for the ICA and ECA, now above the MID, after the BIF. The range for this measurement was between 25 and 32 frames of distance, and the suggested range allows for proper artery differentiation and border definition but varies on patient and side. The ECAt and ICAt, which represent ECA and ICA diameters, were selected so that there is only frame of distance between them; in this manner, the models will have similar artery lengths, allowing for better visualization.

#### 2.6.4. Other Considerations

The measurements of angles and diameters were a semi-automatic process. On the one hand, the 3D Slicer software provided enough automatic tools to obtain the 3D specimen, including boundary of the vessel and circular fit. On the other hand, frames and diameters were obtained manually by selecting the best frame (i.e., the frame with complete geometries) around the landmarks and using the 3D Slicer’s tools to measure diameters. Some sources of error within the manual aspect of the semi-automatic process include determining the specimen position in the sagittal, coronal and transverse planes and finding the exact MID for overall landmarks. Two researchers (P.M. and D.C.) performed independent segmentations and measurements in the first 10 samples for reproducibility purposes following a similar approach than previously reported [27]. Briefly, both researchers independently processed 10 samples following the segmentation processing protocol (2.4 and 2.5) and then determined the landmarks to perform the measurements. Finally, a coefficient of variation (CV) between their independent measurements was calculated, which was lower than 10%.

### 2.7. Statistical Analysis

Data were compiled into a Microsoft^®^ Excel^®^ (for Microsoft 365 MSO (Version 2404 Build 16.0.17531.20152) 64-bit) spreadsheet and then exported into SPSS 29.0 (IBM, Chicago, Il, USA). Data normality was assessed using the Shapiro–Wilk test. Then, a single t-test comparison between sexes for demographic data and a one-way repeated measures, analysis of variance (rmANOVA) where side (left vs. right) is a within-subject factor (each participant has measurements from both sides), and sex (females vs. males) is a between-subject factor (different groups of participants). Finally, post hoc analyses were performed using least square differences (LSDs). Statistical significance was set at alpha = 0.05.

## 3. Results

All variables were found to be normally distributed. The characteristics of the sample are provided in Table 1. Males were taller and heavier and had higher resting systolic blood pressure than females (*p* < 0.05). No differences were observed in age and BMI between sexes. In addition, no atherosclerotic plaque was observed in the subjects’ images.

Even though there was no “side × sex” interaction in any of the anatomical landmarks, there was a trend of a “sex” effect for BIF and ECA (*p* = 0.068 and *p* = 0.069, respectively) (Table 2). This trend is driven by a significant difference in the left BIF (*p* < 0.05) and a solid trend in the left ECA (*p* = 0.05) where males had larger diameters than females.

## 4. Discussion

The purpose of this study was to evaluate the sex-specific anatomical differences in the carotid artery using MRI and a lab-specific imaging processing protocol. This study included young healthy males and females and focused on comparing key anatomical landmarks of the carotid artery. The main findings indicate a trend for larger left bifurcation (BIF) and external carotid artery (ECA) diameters in males, but no more differences between sexes in other diameters or angles.

These findings are noteworthy since larger vessel diameters are associated with reduced endothelial shears stress (ESS), given that ESS is inversely proportional to the diameter of the vessel. This would suggest that males would experience less ESS compared to females; however, previous studies have consistently found no significant differences in ESS between sexes, both at rest and during exercise [4,7]. This apparent contradiction might be attributed to compensatory factors. For example, the current study showed a higher systolic blood pressure observed in males (Table 1). As higher blood pressure elicits higher blood flow velocity, higher blood pressure could counterbalance the effects of the larger diameters, thus normalizing ESS between sexes. Our previous studies have shown similar hemodynamics responses [4,7,8], where males have slightly higher systolic blood pressure and females slightly smaller diameters what showed no differences in ESS at rest or during exercise. These results confirm the alignment with hemodynamic principles and highlight the intricacies of the dynamics between vascular geometry and systemic pressure.

The relationship between carotid artery geometry and ESS is relevant to atherogenesis [3]. On the one hand, increased ESS promotes biochemical signals that regulate vascular tone and structure primarily mediated by mechanotransduction and nitric oxide (NO) metabolism [3,6]. On the other hand, vascular bifurcations elicit complex blood flow patterns, such as disturbed or turbulent flows, where ESS is decreased, which is considered pro-atherogenic [6]. However, turbulent flow can increase ESS in some conditions, like in vascular stenosis [5] or during exercise [4,7], activating beneficial mechanotransduction pathways to prevent or treat atherosclerosis. The measurements of angles and diameters in the BIF region in the current study align with this concept, where the larger male diameters could influence mechanotransduction pathways by altering local shear forces, especially at the bifurcation, influencing the susceptibility and progression of atherosclerosis, particularly in male Hispanics.

Interestingly, while stroke incidence is higher for females across the entire population, in Hispanics, this trend is reversed, with higher stroke rates reported for males [1,2]. Considering that our participants consisted in its majority of Hispanic adults, it is important to contextualize these findings within the demographic characteristics of our sample. Taking this into consideration, the observed anatomical differences, even when combined with the higher blood pressure, may contribute to variations in ESS and, subsequently, the higher stroke risk in this population. However, a larger study including different races, ethnicities, and behaviors (such as nutrition and physical activity) would be needed to determine the reasons why male Hispanics have a higher stroke incidence than female Hispanics.

The geometry of the CCA, ICA, and ECA presented in the current study is similar to previous studies. For example, Goubergrits et al. [28] studied 86 carotid artery specimens from 41 post-mortem subjects. The authors did not compare between sexes; however, they found that the diameters of CCA at BIF was 6.61 ± 1.00 mm, ICA was 4.98 ± 1.08 mm, and ECA was 4.28 ± 0.88 mm. Finally, the reported ICA and ECA mean angles were 33.8° ± 12.2° and 27.8° ± 12.0°, respectively [28]. Our measurements for the BIF compared to the diameters reported for the common carotid artery are in reasonable agreement (Table 2); however, our male sample presents a significantly higher mean diameter. Interestingly, the ICA and ECA diameters and angles in the current study are smaller and larger, respectively, than the post-mortem specimens. These differences could be explained by the samples’ age differences, demographics, and the differences in methodologies.

Using a similar approach to the current study, Thomas et al. studied 50 subjects, 25 young and 25 older adults, to determine carotid artery geometry [29]. The authors found significant differences in bifurcation angle, tortuosity, and diameter ratios between age groups. Even though Thomas et al. used different makers than the current study, they determined a bifurcation angle of 48.5° ± 6.3° in their younger participants, which is around half the BIF angle in the current study (Table 2). Moreover, the BIF angles in the current study are closer to the older adults reported by Thomas et al. [29]. It would be interesting to determine if racial or ethnic factors could be driving these differences.

These contradictory results confirm the diversity of human anatomy, which can impact generalizations. For example, previous approaches have employed computational fluid dynamics (CFD) tools to develop surrogate models that represent the relationship between the geometric parameters of the carotid artery, such as diameters and bifurcation angles, and the flow metrics of interest [25,30]. These efforts have highlighted the importance of observing how the geometric variations that we have reported between males and females could impact the distributions of ESS and vascular outcomes.

The current study is not without imitations. It is important to note that the generated carotid model is carried out by automated processes powered by 3D Slicer, resulting in an approximation of the real carotid. The full design presents defects caused by the patient’s carotid vasodilation due to blood flow, as well as their movement, while being scanned and the difference in thresholds between pixels of the scan. In addition, the sample studied was small and restricted to young, apparently healthy adults, mostly Hispanics. A larger sample, including different demographics, races, and ethnicities, would increase the power of the current results.

## 5. Conclusions

Sex-specific differences for the geometric characteristics of the carotid artery is provided, with males exhibiting larger BIF and ECA diameters compared to females. These variations could provide an explanation for the higher stroke risk observed in specific populations, particularly how Hispanic males have higher risk than females, contrary to other demographics where females have higher stroke rates than males. These findings emphasize the importance of considering ethnicity when evaluating vascular risks.

The advantages of non-invasive imaging procedures, such as MRI, offer practical approaches to the state of vascular research; however, it is not free of limitations, for instance, it relies on automated processes for segmentation.

This study also highlights the necessity of larger and more diverse studies, as well as the potential for heavier computational approaches, for the modeling and understanding of vascular flows in relation to anatomical differences between populations to better comprehend distinct vascular outcomes.

## Figures and Tables

**Table 1:** Participant characteristics.

	Overall (*n* = 20)	Females (*n* = 10)	Males (*n* = 10)	*p*
Age (years) Mean (SD)	26.9 (3.4)	26.3 (2.4)	27.4 (4.2)	0.48
Height (m) Mean (SD)	1.68 (0.10)	1.62 (0.06)	1.73 (0.10)	0.01
Weight (kg) Mean (SD)	68.9 (14.3)	62.2 (10.0)	75.7 (15.1)	0.03
BMI (kg/m^2^) Mean (SD)	24.5 (4.0)	23.6 (3.7)	25.3 (4.3)	0.38
Resting systolic BP (mmHg) Mean (SD)	113 (9)	109 (9)	117 (8)	0.03
Resting diastolic BP (mmHg) Mean (SD)	73 (14)	71 (11)	76 (17)	0.41

BMI: body mass index; BP: blood pressure.

**Table 2:** Anatomical landmarks of both left and right carotid arteries in young males and females.

	Females		Males		Side Effect	Sex Effect	Side × Sex Interaction
	Left (*n* = 10)	Right (*n* = 10)	Left (*n* = 10)	Right (*n* = 10)	ANOVA (F, *p*)	ANOVA (F, *p*)	ANOVA (F, *p*)
MID (mm) Mean (SD)	3.29 (1.02)	3.27 (0.62)	3.52 (0.77)	3.47 (0.52)	0.081 0.779	0.490 0.493	0.012 0.913
BIF (mm) Mean (SD)	6.02 ** (0.64)	6.68 (1.19)	7.20 ** (1.34)	7.26 (1.27)	2.373 0.141	3.770 0.068	1.617 0.220
ICA (mm) Mean (SD)	3.29 (0.39)	3.25 (0.44)	3.61 (0.48)	3.54 (0.56)	0.244 0.627	3.030 0.099	0.010 0.923
ECA (mm) Mean (SD)	3.00 * (0.62)	2.98 (0.34)	3.68 * (1.28)	3.36 (0.50)	0.656 0.429	3.730 0.069	0.518 0.481
BIF angle (°) Mean (SD)	90.3 (11.7)	89.0 (12.0)	88.4 (13.1)	87.4 (11.8)	1.856 0.190	0.106 0.748	0.022 0.883
ICA angle (°) Mean (SD)	37.8 (13.9)	38.5 (13.3)	41.1 (8.2)	40.4 (8.7)	0.001 0.976	0.268 0.611	0.860 0.366
ECA angle (°) Mean (SD)	52.5 (7.7)	50.5 (10.2)	47.3 (7.8)	47.0 (6.6)	1.454 0.244	1.511 0.235	0.685 0.419

MID: specimen mid-point, which represent the larger diameter of the common carotid artery before the bifurcation, BIF: diameter at transition from the bifurcation to ICA and ECA, ICA: internal carotid artery, ECA: external carotid artery; * *p* = 0.05 and ** *p* = 0.02 males vs. females.

## Data Availability

The raw data supporting the conclusions of this article will be made available by the authors upon request.
